# Twist1 expression induced by sunitinib accelerates tumor cell vasculogenic mimicry by increasing the population of CD133^+^ cells in triple-negative breast cancer

**DOI:** 10.1186/1476-4598-13-207

**Published:** 2014-09-08

**Authors:** Danfang Zhang, Baocun Sun, Xiulan Zhao, Yuemei Ma, Ru Ji, Qiang Gu, Xueyi Dong, Jing Li, Fang Liu, Xiaohua Jia, Xue Leng, Chong Zhang, Ran Sun, Jiadong Chi

**Affiliations:** Department of Pathology, Tianjin Medical University, Tianjin, 300070 China; Department of Pathology, General Hospital of Tianjin Medical University, Tianjin, 300070 China; Department of Pathology, Cancer Hospital of Tianjin Medical University, Tianjin, 300060 China; Department of Pathology, Tianjin Medical University and General Hospital and Cancer Hospital, Tianjin, 300070 PR China

**Keywords:** Triple-negative breast cancer, Cancer stem cell, Vasculogenic mimicry, Angiogenesis, Sunitinib, Twist1

## Abstract

**Background:**

Hypoxia induced by antiangiogenic agents is linked to the generation of cancer stem cells (CSCs) and treatment failure through unknown mechanisms. The generation of endothelial cell-independent microcirculation in malignant tumors is defined as tumor cell vasculogenic mimicry (VM). In the present study, we analyzed the effects of an antiangiogenic agent on VM in triple-negative breast cancer (TNBC).

**Methods:**

Microcirculation patterns were detected in patients with TNBC and non-TNBC. Tientsin Albino 2 (TA2) mice engrafted with mouse TNBC cells and nude mice engrafted with human breast cancer cell lines with TNBC or non-TNBC phenotypes were administered sunitinib and analyzed to determine tumor progression, survival, microcirculation, and oxygen concentration. Further, we evaluated the effects of hypoxia induced with CoCl_2_ and the expression levels of the transcription factor Twist1, in the presence or absence of a Twist siRNA, on the population of CD133^+^ cells and VM in TNBC and non-TNBC cells.

**Results:**

VM was detected in 35.8 and 17.8% of patients with TNBC or with non-TNBC, respectively. The growth of tumors in TNBC and non-TNBC-bearing mice was inhibited by sunitinib. The tumors in TA2 mice engrafted with mouse TNBCs and in mice engrafted a human TNBC cell line (MDA-MB-231) regrew after terminating sunitinib administration. However, this effect was not observed in mice engrafted with a non-TNBC tumor cell line. Tumor metastases in sunitinib-treated TA2 mice was accelerated, and the survival of these mice decreased when sunitinib was withdrawn. VM was the major component of the microcirculation in sunitinib-treated mice with TNBC tumors, and the population of CD133^+^ cells increased in hypoxic areas. Hypoxia also induced MDA-MB-231 cells to express Twist1, and CD133^+^ cells present in the MDA-MB-231 cell population induced VM after reoxygenation. Moreover, hypoxia did not induce MDA-MB-231 cells transfected with an sh-Twist1 siRNA cell to form VM and generate CD133^+^ cells. Conversely, hypoxia induced MCF-7 cells transfected with Twist to form VM and generate CD133^+^ cells.

**Conclusions:**

Sunitinib induced hypoxia in TNBCs, and Twist1 expression induced by hypoxia accelerated VM by increasing population of CD133^+^ cells. VM was responsible for the regrowth of TNBCs sunitinib administration was terminated.

**Electronic supplementary material:**

The online version of this article (doi:10.1186/1476-4598-13-207) contains supplementary material, which is available to authorized users.

## Background

Breast cancer is the most frequent malignancy among women worldwide [1]. The status of the expression of the estrogen receptor (ER), progesterone receptor (PR), and human epidermal growth factor receptor 2 (HER2) are the most important prognostic markers for invasive breast cancer [2]. Triple-negative breast cancer (TNBC) cells do not express ER, PR, or HER2 [3]. TNBC accounts for approximately 15–26% of breast cancer cases worldwide [4–7]. The survival of patients with TNBC is shorter compared with that of patients with other breast cancer subtypes because of the unique genotype and clinical behavior of TNBC [3]. TNBCs are more likely to be aggressive and have a higher tendency to metastasize to visceral organs. Patients with TNBC do not benefit from endocrine therapy or from anti-HER2 antibody therapy [8]. Moreover, the chemosensitivity of TNBCs is limited. Despite clinical trials, an efficacious treatment for patients with TNBC is not available [9, 10].

Antiangiogenic agents such as a anti-vascular endothelial growth factor (VEGF) neutralizing antibody (Avastin, bevacizumab) and inhibitors of VEGF receptor tyrosine kinase activity (sorafenib and sunitinib) are key components of front-line combination regimens for the treatment of various human cancers [9–11]. These agents are used to treat non-small cell lung cancer, renal cell cancer, and hepatocellular carcinoma [12–14] and were used to treat metastatic breast cancers in preclinical and clinical studies [15]. However, these and other studies indicate that these therapies may have limited efficacy [16–18]. Although these agents inhibit the growth of primary tumors, the responses are usually temporary, and the overall survival of patients is only modestly increased [19]. Further, when antiangiogenic agents are administered intermittently, for example, sunitinib (4 weeks on and 2 weeks off), tumor regrowth is sometimes observed during drug-free periods or upon termination of treatment [20, 21].

Given the limited effect of such treatments, several clinical trials of sunitinib or bevacizumab to treat breast cancer were terminated. One study reported increased tumor invasiveness and metastasis after using VEGF inhibitors or inactivating VEGF gene expression in mouse models of cancer [22]. These reports suggest that the rationale and prospects of antiangiogenic therapies for breast cancer treatment must be re-evaluated. Because of this, we asked two questions as follows: 1. What is the mechanism of antiangiogenic treatment failure? 2. Is there any difference in the responses to anti-VEGF agents of patients with TNBC or non-TNBC?

In 1999, Maniotis *et al.* reported the discovery of vasculogenic mimicry (VM), a vascularization of malignant tumors [23]. VM channels are formed by tumor cells but not by endothelial cells. VM occurs in many aggressive tumors such as melanoma, inflammatory breast carcinoma, prostate carcinoma, ovarian carcinoma, hepatocellular carcinoma, and gastrointestinal stromal tumors [24–28]. Tumors with VM are more aggressive, and patients have a poorer prognosis than those without VM.

We proved that hypoxia induces VM, and uncovered evidence that cancer stem cells (CSCs) may play an important role in VM [29, 30]. Moreover, administration of antiangiogenic agents induces intratumoral hypoxia, and hypoxia increases the number of CSCs in cell lines derived from glioblastomas and breast cancers [31]. Based on these results, we hypothesized that intratumoral hypoxia induced by antiangiogenic agents accelerates VM channel formation in TNBC by increasing the population of CSCs, which in turn, causes tumor regrowth, metastases, and treatment failure using antiangiogenic agents. This hypothesis is supported by the results of the present study that includes an analysis of human patients with TNBC and non-TNBC as well as studies conducted in *vivo* and in *vitro* using mice that develop spontaneous TNBC and nude mice engrafted with human breast cancer cell lines with TNBC and non-TNBC phenotypes.

## Results

### Pathological and clinical features of TNBC

**Figure 1:**
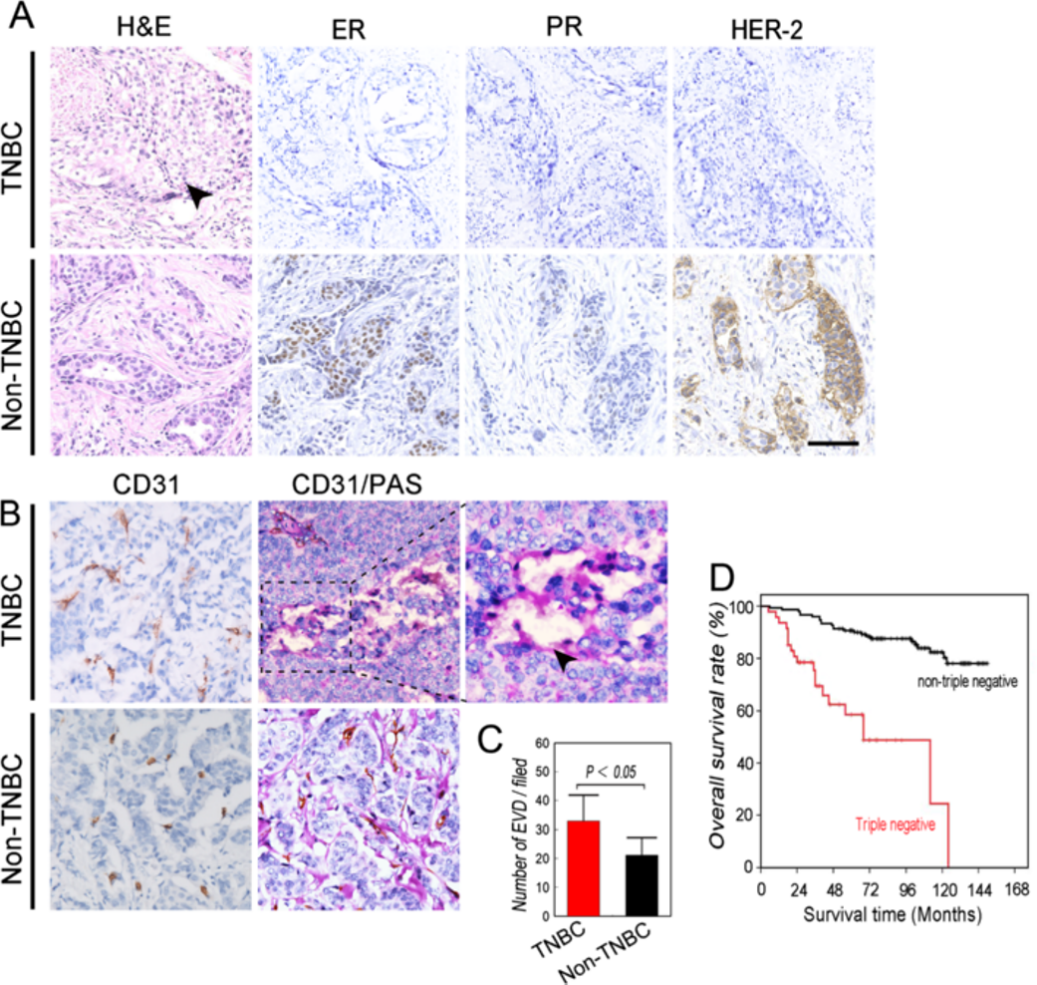
**Characterization of the TNBC and non-TNBC groups.** Patients were diagnosed with TNBC or non-TNBC using immunohistochemical criteria and group accordingly (see Methods section). **A)** H&E staining and IHC analysis of ER, PR, and HER2 expression in tumor sections. Tumor nests comprised poorly differentiated highly mitotic small tumor cells in patients with TNBC, and necrosis was present in the center of the tumor nests (indicated by the black arrow). **B)** IHC analysis of CD31 expression indicates endothelium-dependent vessels (EDV) in sections from patients with TNBC and non-TNBC. CD31/PAS double-staining shows more VM channels in TNBC sections. The arrow indicates a VM channel formed by PAS-positive matrix and tumor cells in a TNBC section. **C)** Comparison of the density of EDVs between groups. **D)** Overall survival of patients with TNBC and non-TNBC. Kaplan–Meier analysis indicates that the prognosis of the TNBC group was poorer (*χ* ^2^ = 7.587, *P* = 0.006). Scale bar = 100 μm. The error bar indicates the standard deviation (SD).

The expression of ER, PR, and HER2 was determined using immunohistochemistry (IHC), and positive samples were assigned a staining index value >1 (see Methods). Among the 174 patients with breast cancer selected for this study, 67 were diagnosed with TNBC (TNBC group) according to lack of detection of ER, PR, and HER2 (Figure 1A). The remaining 107 patients were designated the non-TNBC group. The TNBC group had small, poorly differentiated and highly mitotic tumor cells, and necrosis was present in the center of the tumor nests. Table 1 summarizes the pathological and clinical features of the patients in each group. The median ages at diagnosis of patients in the TNBC and non-TNBC groups were 47 and 51 years, respectively. Approximately 11.9 and 4.7% of these respective patients were <40 years of age (*χ*^2^ = 3.148, *P* = 0.076). Grade III disease was diagnosed in 28.4 and 20.0% of the TNBC and non-TNBC groups, respectively (*χ*^2^ = 5.746, *P* = 0.039). At diagnosis, 62.6 and 32.7% of the TNBC and non-TNBC groups presented with axillary node metastasis (*χ*^2^ = 4.078, *P* = 0.048), respectively, and 17.9% of the TNBC group was diagnosed with clinical stages TNM II or III (*χ*^2^ = 6.347, *P* = 0.050). Distant metastases were present in approximately 14 and 3.8% of the TNBC and non-TNBC groups, respectively (*χ*^2^ = 6.077, *P* = 0.024).

**Table 1:** **Comparison of pathological and clinical features of patients with triple-negative and non-triple-negative breast cancer**

Factors	Triple-negative (%)	Non-triple-negative (%)	***χ*** ^2^	***P***
Age (years)				
<40	8 (11.9)	5 (4.7)	3.148	0.076
≥40	59 (88.1)	102 (95.3)		
Primary tumor size (diameter)				
d<2 cm	17 (25.4)	14 (13.1)	4.659	0.097
2 ≤ d<5 cm	43 (64.2)	76 (71.0)		
d ≥5	7 (10.4)	17 (15.9)		
Grade				
I and II	48 (71.6)	73 (80.0)	5.764	0.039
III	19 (28.4)	34 (20.0)		
Axillary node status				
Negative	25 (37.4)	72 (67.3)	4.078	0.048
Positive	42 (62.6)	35 (32.7)		
TNM stage				
I	13 (19.4)	16 (15.0)	6.374	0.050
II	42 (62.7)	84 (76.6)		
III	12 (17.9)	9 (8.4)		
VM				
No	43 (64.2)	88 (82.2)	5.237	0.002
Yes	24 (35.8)	19 (17.8)		

One hundred eighteen patients were alive at the end of the follow-up period, December 2008. The mean survival of all patients was 120.7 ± 3.9 months. The survival rate of the non-TNBC group was 81.3%, whereas that of the TNBC group was 67.1%. The mean survival times of the non-TNBC and TNBC groups were 128.7 ± 4.15 months and 106.1 ± 7.01 months, respectively (Figure 1D, *χ*^2^ = 7.587, *P* = 0.006).

### Comparison of microcirculation patterns of the TNBC and non-TNBC groups

Using IHC, we analyzed tissues for the expression of CD31 and together with PAS staining was performed to investigate microcirculation patterns. Detection of CD31 expression shows that the number of endothelial vessels in the TNBC group was higher compared with that of the non-TNBC group (Figure 1B and 1C, respectively; *t* = 2.033, *P* = 0.044). VM channels that did not express CD31 but stained with PAS (Figure 1B) were identified in approximately 35.8% of the TNBC group and in 17.8% of the non-TNBC group (Figure 1B, Table 1, *χ*^2^ = 5.327, *P* = 0.002).

### Effects of sunitinib on TA2 mice engrafted with mouse TNBC cells and nude mice engrafted with human cell lines with non-TNBC and TNBC phenotypes

To study the effect of anti-angiogenesis agents on TNBC, TNBC-bearing TA2 mice were treated orally with sunitinib using a clinically relevant schedule. TA2 breast cancers comprise mainly small round cells with a small cytoplasm. These cancers readily metastasize to the lungs, liver, and spleen (Additional file 1: Figure S1A-S1D). Because immunohistochemical analysis of tumors did not detect the expression in tumors of ER-α, PR, or HER-2 (Additional file 1: Figure S1E-S1G), we defined the spontaneous breast cancers of TA2 mice as TNBC. Conversely, the expression p53, PCNA, cyclin D1, and cytokeratin 5/8 was detected in the tumors (Additional file 1: Figure S1H-S1K). Therefore, we used these mice as a model for assessing the effects of sunitinib on TNBC.

**Figure 2:**
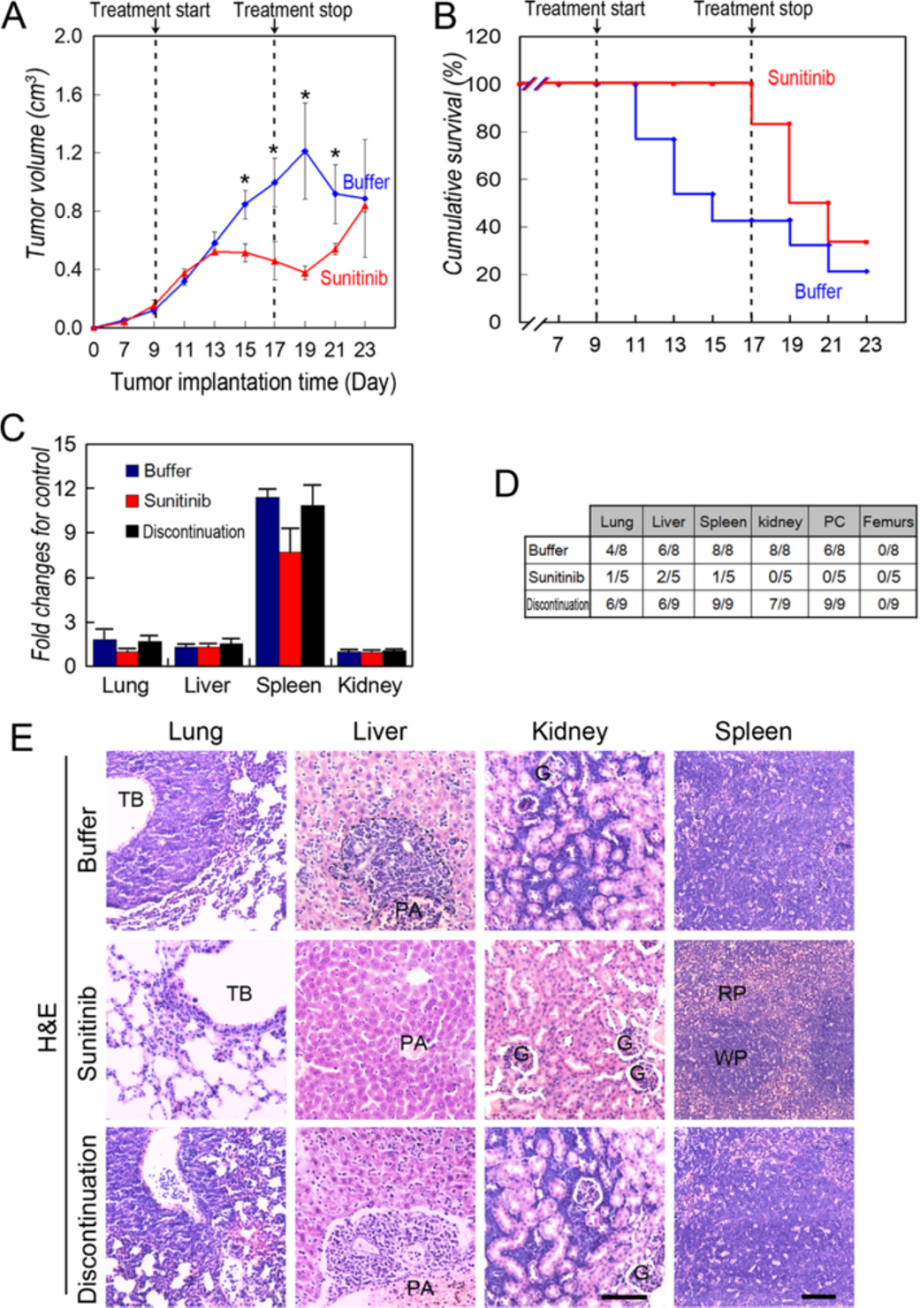
**Effect of sunitinib on the survival, growth, and metastasis of TA2 mice with TNBC. A)** Tumor growth curve of sunitinib- and placebo-treated groups (N = 20 per group). **B)** Survival of groups treated with sunitinib or placebo. **C)** Analysis of the weights of lungs, liver, spleen, and kidneys. **D)** Percentage of mice with metastases in different organs. More metastatic sites were identified in the lungs, liver, spleen, kidneys, and peritoneal cavity of the mice in the control group, and the number of metastases increased when treatment was terminated. **E)** Morphology of metastatic sites in treated and control groups (H&E staining). The scale bar = 100 μm, and the error bar indicates the SD. TB: terminal bronchiole; G: glomerulus; PA: portal area; WB: white pulp; RB: red pulp.

The mice were treated with sunitinib or placebo for 8 days. The tumors grew at a significantly slower rate in the sunitinib-treated mice compared with those administered placebo (Figure 2A). All TA2 mice in the treatment group were alive on day 17, in contrast to <40% of controls (Figure 2B). The sizes of the tumors in mice treated with sunitinib increased after treatment was terminated. Moreover, the survival rate of the treatment group decreased similarly to that of the placebo group after treatment was withdrawn. Moreover, after sunitinib treatment was terminated, the spleens of the placebo-treated and sunitinib-treated mice were significantly enlarged compared with those during treatment with sunitinib (Figures 1A and 2B). Additional metastatic sites were identified in the lung, liver, spleen, kidney, and peritoneal cavity of the control group (Figure 2D). However, bone metastasis was not detected in the animals of all groups (Figure 2D). The organ structures that were disrupted by the metastatic breast cancer cells are shown in Figure 2E.

**Figure 3:**
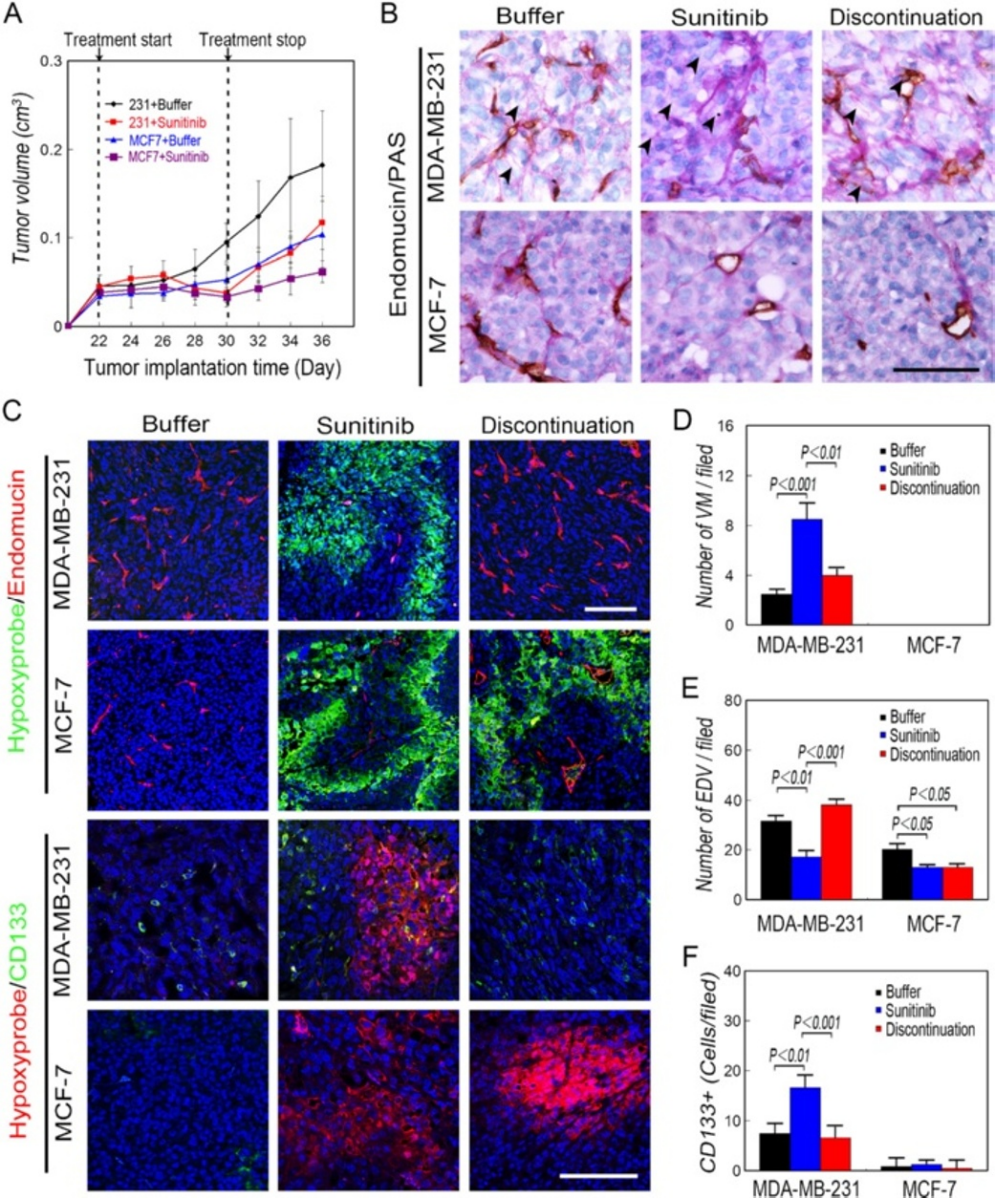
**Effect of sunitinib on nude mice engrafted with MDA-MB-231 and MCF-7 cells. A)** Tumor growth curves of (N = 20 per group). **B)** Microcirculation patterns. The number of EDVs decreased in sunitinib-treated mice with MDA-MB-231 tumors, and more VM channels were observed during and after these mice were treated with sunitinib compared with mice bearing MDA-MB-231-induced tumors that received placebo. The EDVs rebounded after the treatment of mice with MDA-MB-231 tumors. VM channels did not form in mice with MCF-7 tumors. The arrows indicate the VM channels formed by PAS-positive matrix and tumor cells. **C)** Immunofluorescence analysis of endomucin and CD133 expression and Hydroxyprobe analysis of oxygen levels. More Hypoxyprobe-positive cells were observed in the mice with tumors formed by MDA-MB-231 and MCF-7 tumors treated with sunitinib compared with those in the control group. The hypoxic area in tumors formed by MDA-MB-231 cells disappeared when treatment was terminated, and EDVs rebounded upon treatment. Conversely, there was no significant difference between mice with tumors formed by MCF-7 cells during or after treatment with sunitinib. CD133^+^ cells were present in MDA-MB-231 tumors in the center and periphery of the hypoxic area. The number of CD133^+^ cells in MCF-7 tumors did not differ among groups. **D)** Quantification of VM. VM channels were not observed in MCF-7 tumors. **E)** Quantification of EDVs. **F)** Quantification of CD133^+^ cells. More CD133^+^ cells were present in the MDA-MB-231 tumors in mice treated with sunitinib compared with those in other tumors. Scale bar = 100 μm, and the error bar indicates the SD.

We next tested the effects of sunitinib on mice engrafted with either TNBC MDA-MB-231 cells (with the TNBC phenotype) or with MCF-7 cells (with the non-TNBC phenotype). Sunitinib inhibited the growth of tumors induced by MDA-MB-231 or MCF-7 cells (Figure 3A). The tumors formed by MDA-MB-231 cells, but not by MCF-7 cells, regrew when treatment was terminated (Figure 3A). None of the mice in either group died.

### Hypoxia induced by sunitinib accelerates the generation of CSCs and VM in TNBC

To determine the mechanism of the regrowth and metastasis of TNBCs after terminating sunitinib treatment, we performed endomucin/PAS double-staining, immunofluorescence analysis of endomucin and CD133 expression, and Hydroxyprobe analysis of oxygen concentration to determine the microcirculation patterns, the presence of CSC populations in the different treatment groups, and the number of hypoxic cells.

**Figure 4:**
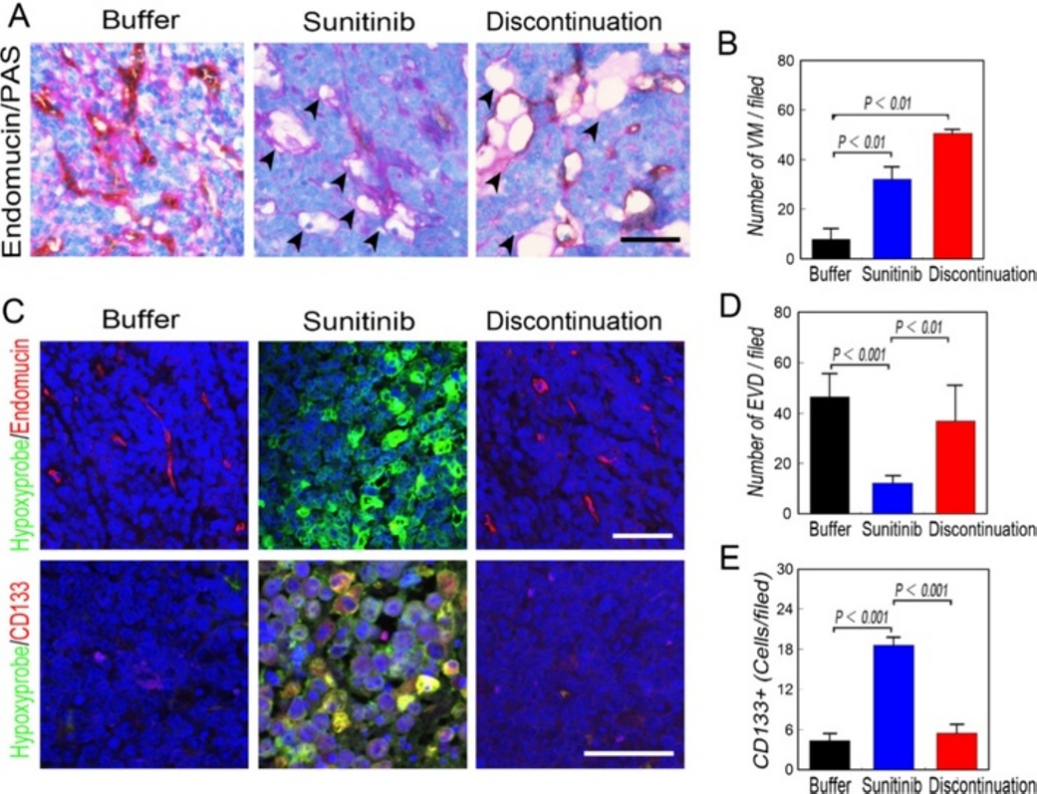
**Sunitinib-induced hypoxia accelerates the generation of CSCs and VM in breast tumors of TA2 mice. A)** Microcirculation patterns of tumors in mice treated with sunitinib. The number of EDVs decreased and more VM channels were present in mice during and after treatment compared with the animals administered placebo. Numerous EDVs rebounded when treatment was discontinued. The arrows indicate the VM channels formed by PAS-positive matrix and tumor cells. **B)** Quantification of VM channels in the treatment groups. VM channels increased during and after sunitinib treatment. **C)** Immunofluorescence analysis of endomucin and CD133 expression and Hydroxyprobe analysis of oxygen levels. More tumor cells were detected using the Hypoxypobe in the tumors of mice treated with sunitinib, and most were CD133^+^. **D)** Quantification of EDVs. **E)** Quantification of CD133^+^ cells in tumors. More CD133^+^ cells were present in the sunitinib-treated tumors. Scale bar = 100 μm, and the error bar indicates the SD.

Endothelial vessels and VM were observed in TA2 tumors and those formed by MDA-MB-231 cells, and VM channels were detected not in tumors formed by MCF-7 cells (Figures 3B and 4A). The number of VM channels in TA2 and MDA-MB-231 tumors increased significantly after sunitinib treatment, and the number of endothelium-dependent vessels decreased (Figures 3B-3E and 4A-4D). Endothelial vessels reappeared in TA2 and MDA-MB-231 tumors after treatment was terminated; however, no significant difference was observed in the epithelial vessels after terminating sunitinib treatment of MCF-7 tumors (Figures 3B-3E and 4A-4D).

Hypoxyprobe analysis confirmed that increased numbers of hypoxic tumor cells were present in TA2 and MDA-MB-231 tumors compared with those of the other groups (Figures 3C and 4C). More CD133^+^ cells were detected in TA2 and MDA-MB-231 tumors compared with the controls and after treatment was terminated. CD133^+^ MDA-MB-231 tumor cells were observed in the center and at the periphery of the hypoxic areas, and the numbers of CD133^+^ MCF-7 tumor cells were similar in all groups (Figures 3C, 3F and 4C, 4E).

### Hypoxia induces the formation of VM-like channels and the generation of MDA-MB-231-CSCs by up-regulating the expression of proteins associated with VM

**Figure 5:**
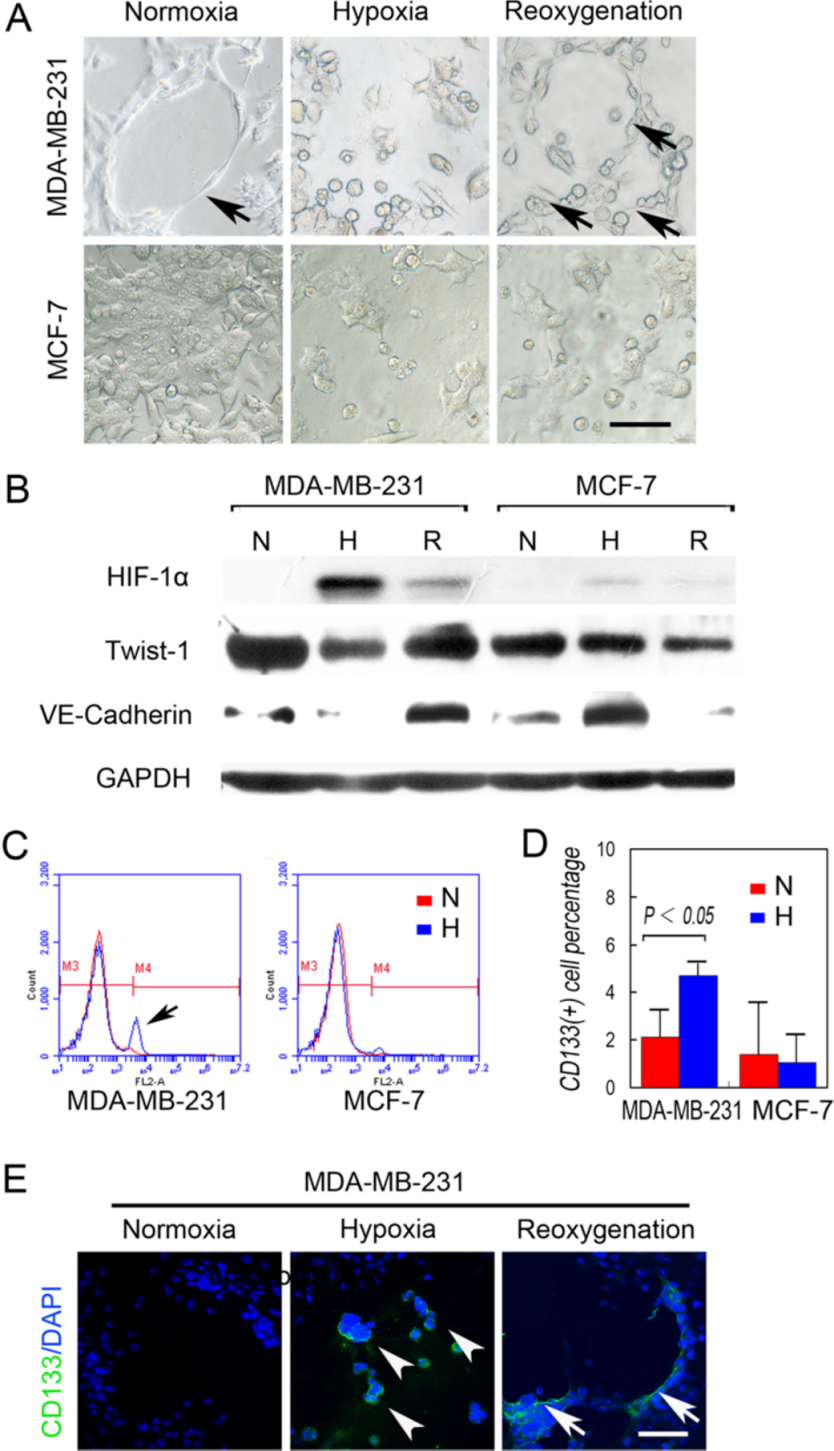
**Hypoxia induces VM-like channel formation by up-regulating the expression of proteins associated with VM and by generating MDA-MB-231 CSCs. A)** Matrigel cell culture under a CoCl_2_-induced hypoxia. MDA-MB-231 cells formed VM-like channels (arrow) on Matrigel under normoxia, and more VM-like channels (arrows) were formed after reoxygenation. In contrast, hypoxia and reoxygenation did not affect VM-like channel formation by MCF-7 cells. **B)** Western blot analysis shows that hypoxia and reoxygenation induced and inhibited HIF-1α expression, respectively, in MDA-MB-231 and MCF-7 cells. However, reoxygenation induced the expression of Twist1 and VE-cadherin only in MDA-MB-231 cells. **C)** Representative FACS analyses of the CD133^+^ populations of MDA-MB-231 and MCF-7 cells in normoxia and hypoxia. The arrow shows the increase in the CD133^+^ population under hypoxia. **D)** Quantification of the CD133^+^ population under normoxia and hypoxia. **E)** MDA-MB-231 cells formed a VM-like channel on Matrigel in normoxia and were CD133^-^. The MDA-MB-231 cells that survived hypoxia were spherical, similar to stem cells and expressed CD133 (arrowheads). The tumor cells that line the VM channels were CD133^+^ (arrows), whereas the cells far from VM were CD133^-^. The scale bar = 100 μm, and the error bar indicates the SD.

To investigate the relationship between hypoxia generated by inhibitors of VEGF signaling and VM of human TNBCs, we induced hypoxic conditions *in vitro* using CoCl_2_. Normoxic MDA-MB-231 cells formed VM-like channels, and the number of these channels increased after CoCl_2_ treatment and reoxygenation (Figure 5A). This result was accompanied by a dynamic change in the expression of HIF-1α expression and an increased Twist1 and VE-cadherin expression (Figure 5B). In contrast to TNBC cells, hypoxia introduced Twist1-independent VE-cadherin up-regulation; however, the VM-like channel formation by MCF-7 cells was not affected by reoxygenation (Figure 5A and 5B).

We next determined the effects of hypoxia on the generation of human breast cancer CSCs and the relationship between hypoxia and VM. FACS analysis indicated that the CD133^+^ population of MDA-MB-231 cells increased significantly after CoCl_2_ treatment, whereas that of MCF-7 cells did not change (Figure 5C and 5D). Immunofluorescence analysis of CD133 expression by cells cultured in Matrigel shows that CD133^+^ MDA-MB-231 cells survived hypoxia and formed a stem-cell sphere. Moreover, we detected CD133 in the tumor cells lining the VM channels but not in distantly located cells (Figure 5E).

### Effects of twist1 on CD133 expression and VM-like channel formation by breast cancer cells

**Figure 6:**
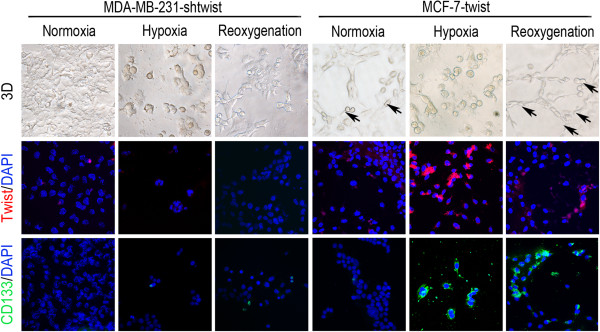
**Effects of Twist1 levels on CD133 expression and VM-like channel formation by breast cancer cells.** MDA-MB-231 cells did not form VM-like channels when Twist1 expression was inhibited under conditions of normoxia, hypoxia, and reoxygenation, and CD133 expression was inhibited as well. When Twist1 expression was up-regulated, MCF-7 cells formed VM channels under conditions of normoxia and reoxygenation. Moreover, the tumor cells that lined the VM channels were CD133^+^. The error bar = 100 μm, and the error bar indicates the SD.

We used siRNA techniques to investigate the role of Twist1 in vasculogenic mimicry induced by TNBCs and the generation of CD133^+^ cells under hypoxia. Twist1 expression was inhibited or increased in MDA-MB-231 transfected with a Twist1-siRNA and MCF-7 cells transfected with Twist1. Transfected MDA-MB-231 cells did not form VM-like channels under normoxia, hypoxia, or reoxygenation, and CD133 expression was inhibited as well (Figure 6). MCF-7 cells that expressed higher levels of Twist1 formed VM channels under conditions of normoxia and reoxygenation. Moreover, the tumor cells lining the VM channels expressed CD133 (Figure 6).

## Discussion

Vasculogenic mimicry occurs in over 10 tumor types [23, 32] that are characterized as highly aggressive, poorly differentiated, and highly metastatic [32, 33]. Therefore, patients with tumors characterized by vasculogenic mimicry have poor outcomes compared with those without VM [24]. Compared with other tumors, TNBCs are larger, higher grade, more aggressive, and they present with lymph node involvement [4, 7, 34]. We found that VM occurs more frequently in patients with TNBC compared with those with non-TNBC, which strongly supports the conclusion that VM indicates poor prognosis.

Hendrix *et al.* proposed that tumor cells with embryonic phenotypes are highly plastic and form VM channels [33]. The genes that express proteins that contribute to the formation of VM channels are specifically expressed by endothelial and hematopoietic stem cells [35, 36]. Recent reports indicate that CSCs may be involved in VM in glioblastomas, breast cancers, and hepatocellular carcinomas [30, 37]. Hepatocellular carcinoma cells that form VM channels express CSC markers such as SOX2 and OCT4 (Sun BC *et al*. unpublished observations). Glioma cancer stem cells enriched in the human glioblastoma cell line U87 form VM channels in xenograft transplantation [37].

Analysis of the gene expression profiles of 587 patients with TNBC shows enrichment of markers specific for stem cells or mesenchymal stem cells [38]. The epithelial-to-mesenchymal transition (EMT) is important in VM. Moreover, genes encoding proteins associated with the EMT are expressed at high levels in this population of patients [39]. Moreover, these results suggest that the gene expression fingerprint of TNBC determines the ability of TNBCs to form VM more efficiently compared with non-TNBCs.

Bevacizumab and sunitinib in combination with cytotoxic drugs were administered in phase III trials of patients with metastatic breast cancer, and bevacizumab was evaluated for treating patients with early-stage breast cancer as a neoadjuvant [40]. The failures of these trials recently provoked several debates regarding the future applications of anti-VEGF agents in breast cancer [20, 41]. Aside from the failure of anti-VEGF agents to treat breast cancer, the results of clinical and preclinical research show that they have limited efficacy for treating hepatocellular carcinoma, rectal cancer, and glioblastoma [14]. These treatment failures may be explained as follows [16, 19]: *(i)* some breast cancers are highly angiogenic and express low levels of VEGF; *(ii)* numerous proangiogenic growth factors such as PLGF, PDGFBB, and bFGF are present and can be up-regulated to drive angiogenesis when the VEGF pathway is inhibited [10]; and *(iii)* antiangiogenic therapy can increase tumor hypoxia, which induces an increase in HIF-1α expression to levels sufficient to activate genes that encode proteins required for the growth, invasion, and metastasis.

In the present study, sunitinib was administered to TA2 mice engrafted with tumors of the TN phenotype that were derived from TA2 spontaneous breast cancers [42]. The growth of primary tumors and metastases were typically inhibited by sunitinib treatment, and the survival of the treated mice increased from 40 to 100%. However, the mice suffered from tumor regrowth and metastases when treatment was suspended, which decreased to those of the control group.

Our previous study reveals “three stages of tumor microcirculation” in melanomas [32]. VM channels, mosaic blood vessels, and endothelial vessels coexist in a malignant tumor and can transform into each other by changes in the tumor microenvironment [29, 43]. Because VM occurs in TNBC, we investigated the microcirculation patterns of the sunitinib-treated tumors and observed numerous VM channels when endothelial vessels were inhibited by sunitinib. After discontinuing treatment, the number of endothelial vessels increased and were linked to the VM channels.

The VEGF signaling pathway is essential in endothelial cell-dependent angiogenesis. However, VM is independent of VEGF [32]. For example, when endothelial vessels are blocked by anti-VEGF agents, VM can be triggered to provide blood to promote tumor growth and metastasis. Moreover, VM is responsible for regenerating the endothelial vessels when treatment is discontinued in this study. These results implicate VM in the failure of standard antiangiogenic therapy to kill aggressive tumors. Therefore, devising strategies that combine standard VEGF-targeted therapies or an endothelium-dependent drug with VM-targeted therapies is attracting considerable interest [44].

A hypoxic tumor microenvironment is the most important inducer of VM [28, 29, 39]. Consistent with these findings, we show here that sunitinib inhibited endothelial vessels, produced additional hypoxic areas in TNBC-bearing TA2 mice, and increased the number of VM channels. These results are consistent with our previous finding that hypoxia of the ischemic back limb promotes VM in the B16 melanoma mouse model. We also found that CoCl_2_-induced hypoxia increased the number of VM-like channels of MDA-MB-231 cells cultured in Matrigel in the present study.

The mechanism responsible for the effects of hypoxia on VM is unknown. The downstream effectors of HIF-1α are associated with angiogenesis, cell proliferation, cell survival, and glucose/iron metabolism. Hypoxia is linked to increased numbers of CSCs in glioblastoma and breast cancer, and CSCs are involved in tumor angiogenesis and VM [31, 45, 46]. The breast cancer cells of TA2 mice expressed increased levels of CD133 under hypoxia after sunitinib treatment. Further, hypoxia induced an increase in the population of CD133^+^ MDA-MB-231 cells in *vivo*. Moreover, stem cells that formed spheres survived and expressed CD133 under hypoxia. Only the CD133^+^ cells formed VM channels in Matrigel after reoxygenation, suggesting that hypoxia accelerates VM by stimulating the CSC population.

We found that the EMT factor Twist1 induced the expression of VE-cadherin to promote VM in hepatocellular carcinoma [28]. Hypoxia induces an EMT-like phenotype in cancer cells [47], and HIF-1α regulates the expression of Twist1 by binding directly to the hypoxia-response element (HRE) in the proximal promoter of Twist1. EMT can induce stem-cell generation by normal and tumor cells [48, 49]. We show here that MDA-MB-231 cells with up-regulated Twist1 expression increased the CSC population after reoxygenation. Therefore, more VM channels were generated by cells cultured in Matrigel.

Hypoxia did not significantly affect the CSC population in cultures of MCF-7 cells, and Twist1 expression was down-regulated after reoxygenation. Inhibiting Twist1 expression by MDA-MB-231 cells caused the loss of VM channels in Matrigel and decreased the number of CD133^+^ cells in hypoxic cultures. MCF-7 cells that expressed Twist1 gained the ability to form VM channels and generate CD133^+^ cells. These results highlight the complexity of the mechanism that regulates the EMT and the biology of CSCs. Human mammary epithelial cells are transformed by Twist1 and snail, and they exhibit the characteristics of CSCs, including the formation of mammospheres, colonies in soft agar, and tumors in *vivo* [49]. For example, Borgna *et al.* found that MCF-7 cells gain mesenchymal features by enriching for CSCs in short-term mammosphere culture [50]. Whether the EMT regulates CSCs or CSCs regulate the EMT is unknown.

Therefore, hypoxia induced by sunitinib accelerates VM by increasing Twsit1 expression and the population of CSCs in TNBC. This finding may explain the inefficacy of antiangiogenic agents in certain breast cancers. Most important, Twist1 and related signal transduction pathways may serve as targets for treating TNBC.

## Conclusions

Sunitinib induced hypoxia in TNBCs, and Twist1 expression induced by hypoxia accelerated VM by increasing the size of the population of CD133^+^ cells. VM was responsible for the regrowth of TNBCs sunitinib administration was terminated.

## Methods

### Reagents and cell culture

The primary antibodies used in this study are listed in Additional file 2: Table S1. All secondary antibodies were purchased from Zhongshan Golden Bridge Biotechnology Co., Ltd. (Beijing, China). Sunitinib malate (S-8803) was purchased from LC Laboratories (MA, USA). The Hypoxyprobe-1 Kit (*HP1-1000Kit*) was purchased from Hypoxyprobe, Inc. (MA, USA). The human breast cancer cell lines MCF-7 and MDA-MB-231 and breast cancer cells of TA2 mice were cultured in RPMI-1640 medium with 10% FBS, 4 mM L-glutamine, and 1% penicillin-streptomycin. Matrigel (BD Bioscience, NY, USA) was diluted with RPMI-1640 medium.

### Patient samples

The Tianjin General Hospital Ethics Committee approved the studies of humans. The patients were informed of the aims, methods, and other details of the present study. All clinical investigations were conducted according to the principles stated in the Declaration of Helsinki. We collected samples from 174 patients with breast cancer with detailed pathological and clinical information. All patients underwent surgery and chemotherapy in Tianjin General Hospital from 1997 to 2004. The median age of the patients was 51.0 years (range, 31–74 years). All patients had invasive breast cancer, and axillary node metastases were present in 76. The diameter of the primary tumor in 31 patients was <2 cm and >5 cm in 24 patients. The follow-up period started at the time of the surgery and ended in December 2008.

### Tissue microarrays and scoring methods

Formalin-fixed, paraffin-embedded tissues from the patients were analyzed after H&E staining. Specific tissue samples were chosen to create tissue microarrays with 1-mm cores (1.5-mm between cores). Tissue microarrays were analyzed using IHC according to a standard protocol [43]. Protein expression levels were quantified according to the method of Sun *et al.* [43]. Staining was scored as follows; 0 = undetectable, 1 = weak, 2 = moderate, and 3 = strong. The number of positive cells out of 100 tumor cells per field was visually evaluated and scored as follows: 0 < 10% positive, 1 < 25%, 2 < 50%, and 3 > 50%. The staining index or the sum of the staining intensity and the positive-cell score were used to determine the result for each sample. A sample was defined as positive when the staining index was >1. VM and endothelial vessels were counted at 400× magnification, and the score for each sample was defined as the average of 10 fields-of-view.

### TA2 and nude mouse models of TNBC

Tianjin Medical University Ethics Committee approved the protocols for using animals. All steps were carefully administered to protect the welfare of the animals and prevent their suffering. The Tientsin Albino 2 (TA2) mice were provided by the Animal Center of Tianjin Medical University. TA2 mice develop spontaneous breast cancer with the TN phenotype at high incidence (showed in Additional file 3), and we used these tumors to induce tumors in TA2 mice [42]. Nude mice were purchased from Beijing HFK Bioscience Company. Approximately 1 × 10^6^ TA2 breast cancer, MDA-MB-231, and MCF-7 cells were injected subcutaneously into the backs of 6-week-old female nude mice (N = 20 per group, respectively). Tumors were measured every 2 days, and tumor size was calculated using a standard formula (length × width^2^ × 0.52). The TA2 mice with breast cancer were administered sunitinib daily when the tumor reached 0.2 cm^3^. Sunitinib was administered to nude mice when the size of tumors induced by engrafted MDA-MB-231 and MCF-7 cells was approximately 0.05 cm^3^. Sunitinib (60 mg/kg) was administered orally for 8 days, and distilled water was used as the placebo. Survival was closely monitored daily at least three times. All surviving mice were sacrificed 1 week after treatment was terminated. Pimonidazole-HCl was injected intraperitoneally (60 mg/kg) 60 min before the mice were sacrificed. The primary tumors and metastatic sites in the peritoneal cavity, lungs, liver, spleen, kidneys, and femurs were collected, weighed, and fixed with 4% paraformaldehyde (PFA). All organs and tumors were embedded in paraffin, and 5-μm-thick sections were prepared.

### Hypoxic cell culture in *vivo*

MDA-MB-231 and MCF-7 cells were seeded into 96-well plates or on Matrigel-coated slides. Cells were treated with 40 μg/ml CoCl_2_ in cell culture medium for 30 h, and then the hypoxic medium was removed and replaced with normal medium. After 40 h, the cells or slides were harvested, and images were acquired using an inverted microscope (ECLIPSE TS100, Nikon).

### Western blotting

HIF-1α, Twist 1, and VE-cadherin expression was analyzed using western blotting. Lysates were prepared using a buffer containing 1% SDS, 10 Mm Tris–HCl, pH 7.6, 20-μg/ml aprotinin, 20-μg/ml leupeptin, and 1-mM AEBSF. The protein concentration of lysates was measured using the Bradford method. Approximately 20 μg of protein was separated on an 8% SDS-PAGE gel and electroblotted onto a PVDF membrane. After blocking with 5% fat-free milk in TBS-Tween overnight, the membrane was incubated with primary antibodies overnight at 4°C. After washing with TBS-Tween three times, the membrane was labeled with horseradish peroxidase-conjugated anti-goat IgG (1:1,000) for 1 h at room temperature (RT). Blots were developed using a DAB kit, GAPHD was used as an internal control, and the bands were analyzed using a gel imaging system (Kodak).

### Fluorescence-activated cell sorting (FACS) analysis

Suspensions of MDA-MB-231 and MCF-7 cells were fixed in 75% cold ethanol, and 10^6^ cells were incubated with anti-CD133-PE antibody solution or isotype control on ice for 15 min before they were washed, resuspended in staining buffer (2% fetal calf serum in PBS), and analyzed using a FACS Accuri C6 (BD Biosciences). Gates were set with isotype controls for each cell so that <1% of the cell population was false-positive. The labeled cells were then analyzed (10,000 events).

### Immunohistochemical and immunofluorescence assays of formalin-fixed, paraffin-embedded tissues

Formalin-fixed, paraffin-embedded tissues were sectioned, dewaxed, and rehydrated using graded concentrations of alcohol. Endogenous peroxidase was blocked using 5% goat serum at RT for 10 min. The sections were heated in a microwave oven in citrate buffer for 20 min. The slides were incubated with primary antibodies overnight at 4°C, washed with PBS, and individually incubated with biotin-labeled or FITC-labeled secondary antibodies. The color was developed using DAB. The sections were counterstained with hematoxylin or DAPI and observed using a microscope (80i, Nikon).

### Immunohistochemical detection of CD31 and periodic acid Schiff (PAS) double-staining

After immunohistochemical analysis of sections for CD31 expression, the sections were exposed to 1% sodium periodate for 10 min, washed for 5 min in distilled water, and then incubated for 15 min with PAS at 37°C. The sections were counterstained with hematoxylin and observed using a microscope (80i, Nikon).

### Immunofluorescence analysis of cells cultured on Matrigel

MDA-MB-231 and MCF-7 cells cultured on Matrigel-coated slides were washed with PBS twice, permeabilized, and fixed in 2% PFA and 0.1% Triton X100 in PBS buffer at 4°C for 30 min. The slides were then washed three times with PBS and incubated with 10% goat serum in PBS. The cells were then incubated with the primary antibodies at 4°C overnight, washed three times with PBS containing 0.1% Tween-20 for 15 min, and incubated with the secondary antibodies for 2 h at RT. The slides were washed with PBS and mounted using a slow-fade Light Anti-fade Kit (Zhongshan Golden Bridge). All matched samples were photographed using a confocal laser scanning microscope (A1, Nikon).

### Expression plasmids and twist1 gene silencing

A full-length Twist1 complementary cDNA was amplified using PCR from a library of normal human embryo cDNA digested with XhoI/EcoRI and subcloned into pcDNA3.1 vectors [28]. The constructs were confirmed by DNA sequencing. A small interfering RNA (siRNA) kit (pGP-Twist1-shRNA) was purchased from GenePharm (Shanghai, China). The target sequence (5′-AAGCTGAGCAAGATTCAGACC-3′ [siTwist1 nucleotides 505–525]) was used to inhibit Twist1 expression in vitro [28]. A nonsilencing siRNA sequence (target sequence 5′-AATTCTCCGAACGTGTCACGT-3′) was used as a negative control.

### Statistical analysis

SPSS version 11.0 (Chicago, IL, USA) was used to evaluate the data. The *χ*^2^ test was performed to assess the pathological and clinical characteristics of the TNBC and non-TNBC groups. The survival of these two groups was evaluated using Kaplan–Meier analysis. The two-tailed Student *t* test was performed to compare the endothelial vessels of the human breast cancers, tumor growth, metastasis, and CD133^+^ cells between groups. The survival rate of the tumor-bearing mice was evaluated using Kaplan–Meier analysis. Statistical significance was defined as *P* < 0.05.

## Electronic supplementary material

Additional file 1: Figure S1: Morphologic characteristics and phenotype of TA2 breast cancer. **(A)** Spontaneous breast cancers in TA2 mice are mostly composed of poorly differentiated cells and form various tumor nests separated by well-developed stroma. Necrosis (arrow) is frequently found in the center of the tumor. **(B)** Metastatic tumor nodule in the lung. **(C)** Metastatic sites in the liver. **(D)** Metastatic sites in the spleen. **(E)**, **(F)**, and **(G)** show that TA2 breast cancer cells are negative for ER α, PR, and HER-2. **(H)** Moderate expression of p53 is identified in tumor cells. **(I)** Expression of cyclin D1 is detected in TA2 breast cancer. **(J)** PCNA expressed in TA2 breast cancer. **(K)** Expression of Cytokeratin 5/8, a myoepithelium marker, is found in tumor cells. Ruler is 100 μm. (TIFF 11 MB)

Additional file 2: Table S1: Primary antibodies used in this study. (DOC 40 KB)

Additional file 3: **Supplementary data.** (DOC 32 KB)
