# Supplementary figures and images for: Twist1 expression induced by sunitinib accelerates tumor cell vasculogenic mimicry by increasing the population of CD133+ cells in triple-negative breast cancer
Source: Mol Cancer. 2014 Sep 8;13:207. doi: 10.1186/1476-4598-13-207 (PMC4168051; doi:10.1186/1476-4598-13-207)

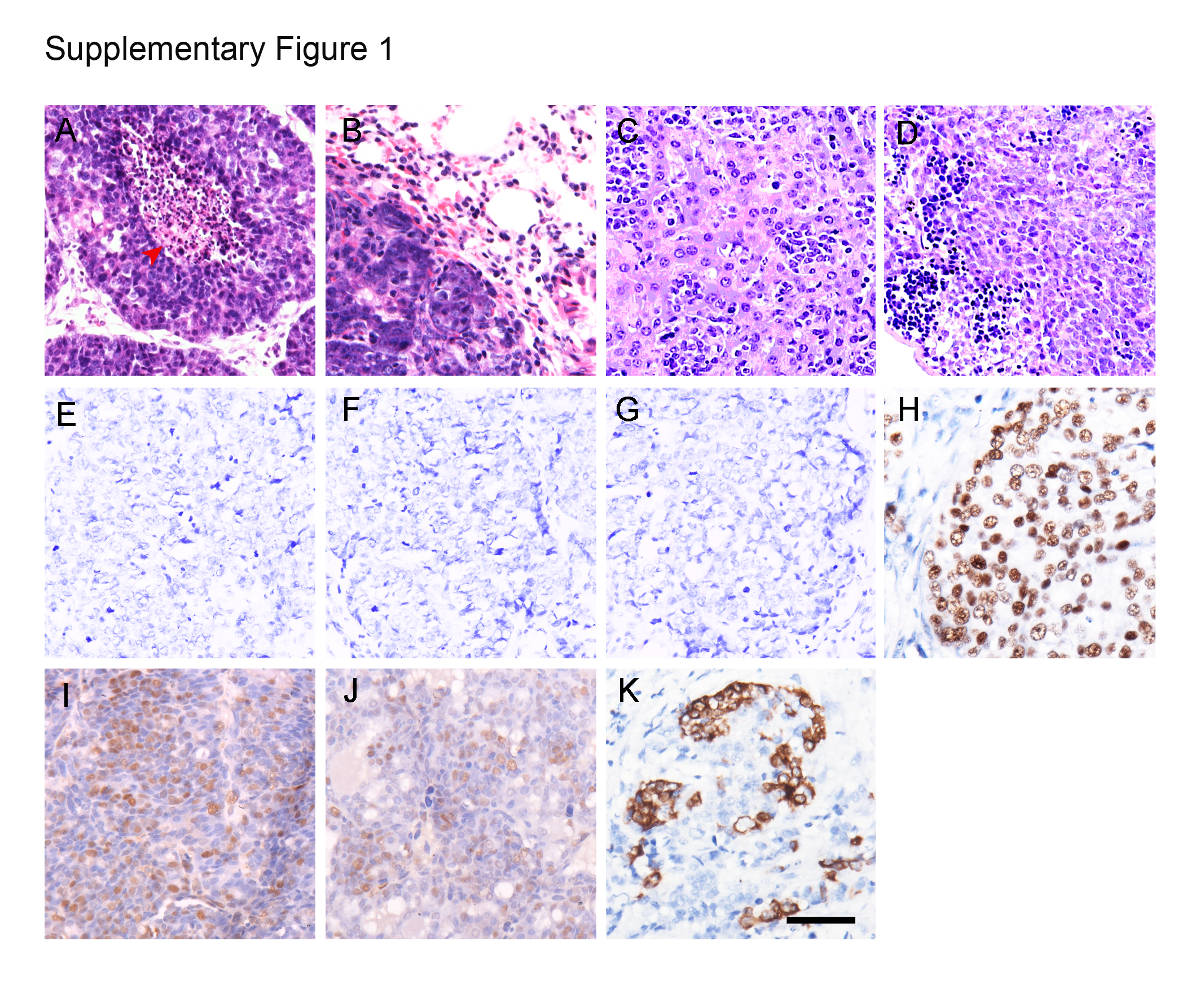

Supplement: Supplementary file 1 — Additional file 1: Figure S1: Morphologic characteristics and phenotype of TA2 breast cancer. (A) Spontaneous breast cancers in TA2 mice are mostly composed of poorly differentiated cells and form various tumor nests separated by well-developed stroma. Necrosis (arrow) is frequently found in the center of the tumor. (B) Metastatic tumor nodule in the lung. (C) Metastatic sites in the liver. (D) Metastatic sites in the spleen. (E), (F), and (G) show that TA2 breast cancer cells are negative for ER α, PR, and HER-2. (H) Moderate expression of p53 is identified in tumor cells. (I) Expression of cyclin D1 is detected in TA2 breast cancer. (J) PCNA expressed in TA2 breast cancer. (K) Expression of Cytokeratin 5/8, a myoepithelium marker, is found in tumor cells. Ruler is 100 μm. (TIFF 11 MB) [file 12943_2014_1407_MOESM1_ESM.tiff]
