# Supplementary material for: Twist1 expression induced by sunitinib accelerates tumor cell vasculogenic mimicry by increasing the population of CD133+ cells in triple-negative breast cancer
Source: Mol Cancer. 2014 Sep 8;13:207. doi: 10.1186/1476-4598-13-207 (PMC4168051; doi:10.1186/1476-4598-13-207)
Supplement: Supplementary file 2 — Additional file 2: Table S1: Primary antibodies used in this study. (DOC 40 KB) [file 12943_2014_1407_MOESM2_ESM.doc]

Additional file 2: Table S1 Primary antibodies used in this study

| Antibody | Source | NO. | Company | Dilution | Application |
| --- | --- | --- | --- | --- | --- |
| ER | Mouse | ZM-0104; | Zhongshan Golden Bridge | ready-to-use | IHC |
| PR | Rabbit | C-20, sc-539 | Zhongshan Golden Bridge | 1:100 | IHC |
| HER2 | Mouse | ZM-0041 | Zhongshan Golden Bridge | ready-to-use | IHC |
| CD31 | Mouse | ZM-0044 | Zhongshan Golden Bridge | ready-to-use | CD31/PAS double staining |
| HIF-1α | Mouse | NB100-105 | Novus | 1:50 | Western blot |
| Twist 1 | Rabbit | Sc-15393 | Santa cruz | 1:200 | Western blot |
| VE-cadherin | Rat | 33168 | abcam | 1:200 | Western blot |
| GAPDH | Rat | Zs-257780 | Zhongshan Golden Bridge | 1:500 | Western blot |
| Endomucin | Rat | 11-5851-80 | eBioscience | 1:400 | Endomucin/PAS double staining |
| CD133 | Rat | MB9-3G8 | Miltenyi Biotec | 1:30 | IF |
| CD133-PE | Mouse | 293C3 | Miltenyi Biotec | 1:20 | FACS, IF |
