# Supplementary material for: Twist1 expression induced by sunitinib accelerates tumor cell vasculogenic mimicry by increasing the population of CD133+ cells in triple-negative breast cancer
Source: Mol Cancer. 2014 Sep 8;13:207. doi: 10.1186/1476-4598-13-207 (PMC4168051; doi:10.1186/1476-4598-13-207)
Supplement: Supplementary file 3 — Additional file 3: Supplementary data. (DOC 32 KB) [file 12943_2014_1407_MOESM3_ESM.doc]

**Supplementary data**

**Materials and Methods**

**Animals**

Ninety female TA2 mice with spontaneous breast cancer were provided by the Animal Center of Tianjin Medical University. All animal work was performed according to protocol approved by the Institutional Animal Care and Use Committee. Complete gross and histopathological evaluations were conducted. After euthanasia, mammary tumors and organs (liver, lungs, and spleen) were collected and fixed in 10% formalin. Formalin-fixed and paraffin-embedded tissues were cut at 5 μm thicknesses, stained with H&E following standard procedure, and examined under a light microscope.

**Immunohistochemical staining**

Paraffin-embedded sections were immunohistochemically stained. Rabbit polyclonal antibodies, including p53 (FL-393), cyclin D1 (H-295), PCNA (FL-261), ERα (MC-20), PR (C-20), mouse monoclonal Neu antibody (F-11), Cytokeratin 5/8 (5F295), and secondary anti mouse antibody (KIT-9701) were purchased from Zhongshan Goldenbridge Biotechnology Co., Ltd. (Beijing, China). The sections were deparaffinized in xylene and rehydrated through graded alcohols to water. Endogenous peroxidase was blocked with 3% hydrogen peroxide in 50% methanol for 10 min at room temperature. After rehydrating, the sections were washed with PBS and pretreated with citrate buffer (0.01 M citric acid, pH 6.0) for 20 min at 100 °C. After rinsing with PBS, slides were incubated with primary polyclonal antibodies overnight at 4 °C. Visualization was performed using diaminobenzidine (DAB). Appropriate positive and negative controls were included.

**Results**

**Morphologic characteristics of TA2 breast cancer**

All spontaneous breast cancers in TA2 mice were mostly composed of small round cells with small cytoplasms that formed various tumor nests separated by well-developed stroma. The center of the tumor nest had masses of undifferentiated cells, many of which were undergoing mitosis. Some tumor cells, especially in the periphery of the tumor, may differentiate into acinar, glandular, and papillary patterns. Acinar tumors were characterized by ductules lined with single or multilayered epithelium and surrounded by dense stroma. The basal layer of the myoepithelium may be maintained. Glandular mammary tumor showed secretory activity, and pink secretions were observed in the glandular cavity. Necrosis was frequently found in the center of the tumor (Figure S1A). Figure S1B, S1C, and S1D showed the metastatic sites in the lung, liver, and spleen, respectively.

Phenotype of TA2 breast cancer

Breast cancer from TA2 mice was triple-negative. Several tumor cells expressed ER α and PR in all mice suffering from spontaneous breast cancer (Figure S1E and S1F). Most breast cancer cells were negative for HER-2 staining (Figure S1G). Moderate expression of p53 was identified in tumor cells (Figure S1H). Expression of PCNA and cyclin D1 were high in breast cancer cells, revealing that breast cancer had a higher proliferation activity (Figure S1I and S1J). Expression of Cytokeratin 5/8, which is a myoepithelium marker, was found in tumor cells (Figure S1K).

**Figure Legends**

Supplementary Figure 1. Morphologic characteristics and phenotype of TA2 breast cancer. (A) Spontaneous breast cancers in TA2 mice are mostly composed of poorly differentiated cells and form various tumor nests separated by well-developed stroma. Necrosis (arrow) is frequently found in the center of the tumor. (B) Metastatic tumor nodule in the lung. (C) Metastatic sites in the liver. (D) Metastatic sites in the spleen. (E), (F), and (G) show that TA2 breast cancer cells are negative for ER α, PR, and HER-2. (H) Moderate expression of p53 is identified in tumor cells. (I) Expression of cyclin D1 is detected in TA2 breast cancer. (J) PCNA expressed in TA2 breast cancer. (K) Expression of Cytokeratin 5/8, a myoepithelium marker, is found in tumor cells. Ruler is 100 μm.
